# Late toxicity and five year outcomes after high-dose-rate brachytherapy as a monotherapy for localized prostate cancer

**DOI:** 10.1186/1748-717X-9-122

**Published:** 2014-05-28

**Authors:** Pirus Ghadjar, Sebastian L Oesch, Cyrill A Rentsch, Bernhard Isaak, Nikola Cihoric, Peter Manser, George N Thalmann, Daniel M Aebersold

**Affiliations:** 1 Department of Radiation Oncology, Charité Universitätsmedizin Berlin, Augustenburger Platz 1, Berlin 13353, Germany; 2 Department of Radiation Oncology with Division of Medical Radiation Physics, Bern University Hospital, Inselspital, Bern, Freiburgstrasse, Bern 3010, Switzerland; 3 Department of Urology, University of Basel, Spitalstrasse 21, Basel 4031, Switzerland; 4 Department of Urology, Bern University Hospital, Inselspital, Bern, Freiburgstrasse, Bern 3010, Switzerland

**Keywords:** Urogenital abnormalities, Radiotherapy, Toxicity, Prostatic neoplasms, Brachytherapy

## Abstract

**Background:**

To determine the 5-year outcome after high-dose-rate brachytherapy (HDR-BT) as a monotherapy.

**Methods:**

Between 10/2003 and 06/2006, 36 patients with low (28) and intermediate (8) risk prostate cancer were treated by HDR-BT monotherapy. All patients received one implant and 4 fractions of 9.5 Gy within 48 hours for a total prescribed dose (PD) of 38 Gy. Five patients received concomitant androgen deprivation therapy (ADT). Toxicity was scored according to the common terminology criteria for adverse events from the National Cancer Institute (CTCAE) version 3.0. Biochemical recurrence was defined according to the Phoenix criteria and analyzed using the Kaplan Meier method. Predictors for late grade 3 GU toxicity were analyzed using univariate and multivariate Cox regression analyses.

**Results:**

The median follow-up was 6.9 years (range, 1.5-8.0 years). Late grade 2 and 3 genitourinary (GU) toxicity was observed in 10 (28%) and 7 (19%) patients, respectively. The actuarial proportion of patients with late grade 3 GU toxicity at 5 years was 17.7%. Late grade 2 and 3 gastrointestinal (GI) toxicities were not observed. The crude erectile function preservation rate in patients without ADT was 75%. The 5 year biochemical recurrence-free survival (bRFS) rate was 97%. Late grade 3 GU toxicity was associated with the urethral volume (*p* = 0.001) and the urethral V_120_ (urethral volume receiving ≥120% of the PD; *p* = 0.0005) after multivariate Cox regression.

**Conclusions:**

After HDR-BT monotherapy late grade 3 GU was observed relatively frequently and was associated with the urethral V_120_. GI toxicity was negligible, the erectile function preservation rate and the bRFS rate was excellent.

## Background

High-dose-rate brachytherapy (HDR-BT) offers the direct application of high doses to the prostate while sparing the surrounding bladder and rectum. It has also advantages over the permanent seed implantation (seeds or low-dose rate brachytherapy) regarding radiation safety and HDR-BT thus gained popularity both as a boost combined with additional external beam radiotherapy or as a monotherapy for localized prostate cancer.

Several groups have described their HDR-BT monotherapy experience [1-9], however less is known about the outcomes after longer follow-up. We therefore performed an up-date of our previously reported HDR-BT monotherapy cohort [9].

## Methods

### Patient selection and characteristics

A total of 41 patients with histologically proven adenocarcinoma of the prostate treated by HDR-BT as a monotherapy were retrospectively analyzed. The pre-treatment staging included a complete history, physical examination, digital rectal examination, transrectal ultrasound (TRUS) of the prostate, biopsy with specification of the Gleason score, pre-treatment prostate-specific antigen (PSA) level, computed tomography (CT) and/or magnetic resonance imaging (MRI) of the abdomen and pelvis as previously reported [9]. In the presence of bone pain a bone scan was performed. The indication for HDR-BT as a monotherapy was limited to low and intermediate risk prostate cancer, similar to criteria for low-dose-rate (LDR) seeds brachytherapy. Risk of recurrence was determined according to the National Comprehensive Cancer Network practice guidelines in oncology (http://www.nccn.org): Low risk being T1-T2a, Gleason score ≤6, PSA <10 ng/mL and intermediate risk being T2b-T2c or, Gleason score 7 or PSA 10–20 ng/mL. Patients with prior pelvic radiation (n = 1) and genitourinary (GU) morbidity ≥ grade 2 prior to radiation (n = 4) were excluded from further analysis. The remaining 36 patients, treated between October 2003 and June 2006, comprised the study population.

This study was approved by the institutional ethics committee of the Bern University Hospital.

### Androgen deprivation therapy

A total of 5 patients (14%) received androgen deprivation therapy (ADT) of which 3 were low and 2 were intermediate risk patients. ADT was initiated in all 5 patients by the referring urologist at their descretion for concommittant treatment. ADT was always given before and during brachytherapy, with a median total duration of 7 months (range, 3–33 months). One patient received combined androgen blockade consisting of antiandrogen and a gonadotropin releasing hormone analogue. Four patients received antiandrogen monotherapy.

### Brachytherapy implant

**Figure 1:**
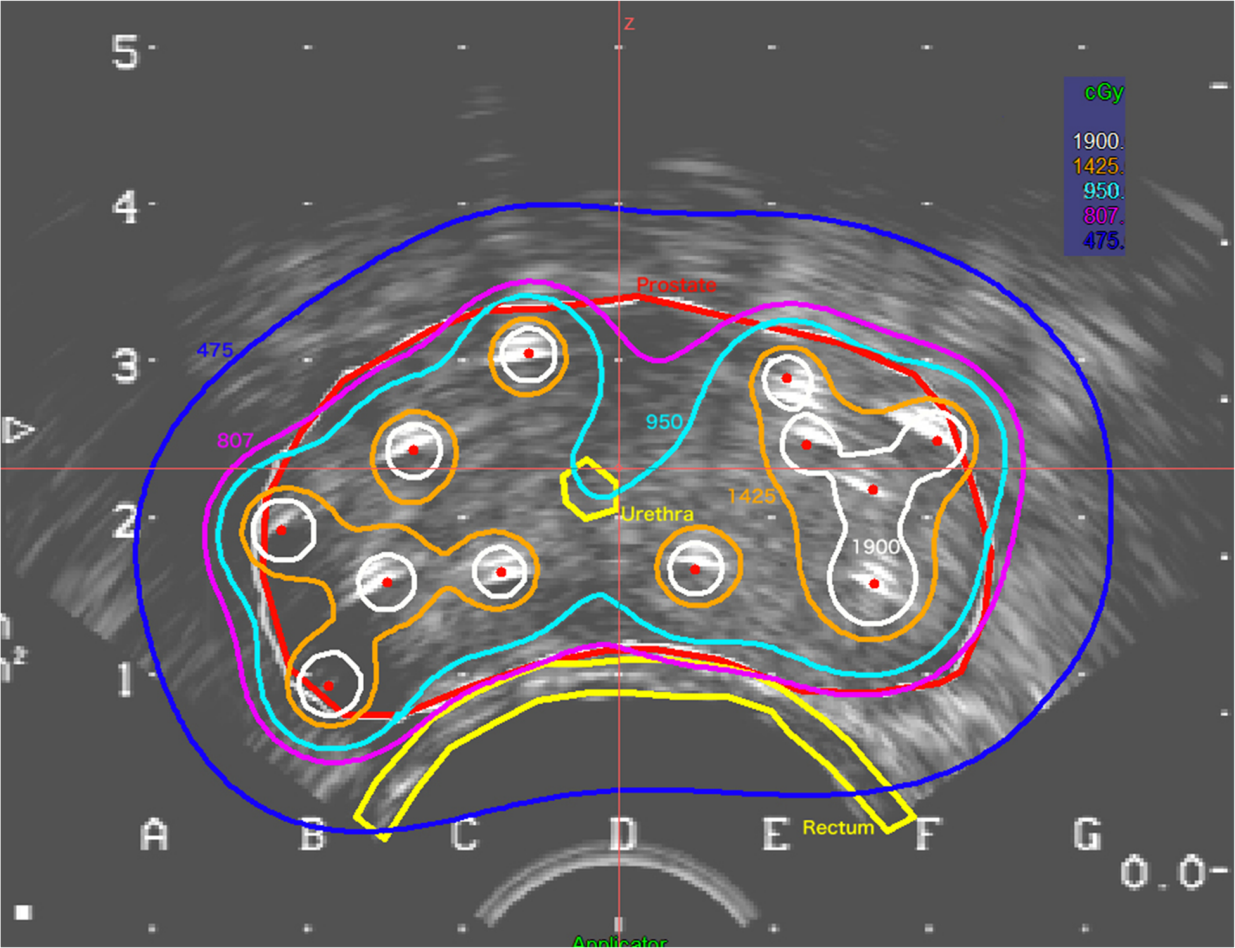
**A typical ultrasound image of the applicator geometry used in HDR-BT.** The red line represents the prostate or the planning target volume (PTV), respectively. Urethra and anterior rectal wall are depicted in yellow. The isodose lines are 50% = dark blue; 85% = purple; 100% = light blue; 150% = orange; 200% = white.

All patients received one implant and four separate fractions of HDR-B, with a minimal interval of 6 hours delivered within 48 hours. Fraction dose was 9.5 Gy, the total prescribed dose (PD) was 38 Gy, as previously described [9]. For the implantation of the applicators, patients underwent spinal anesthesia and were positioned in lithotomy position. The applicators were implanted transperineally by an urologist under real-time TRUS guidance using a template for parallel needle insertion. Patients received a variable number of needles, median 13 (range, 10–18) depending on the prostate size and configuration. Axial cross sections were acquired in 5 mm steps and transferred to the Plato® treatment planning software V14.2. (Nucletron, an Elektra company (Elektra AB, Stockholm, Sweden)). Dose optimization was done on the reconstructed applicator geometry using dose point and manual optimization algorithms. A typical ultrasound image of the applicator geometry is provided in Figure 1. Before radiation, patients received 2–3 gold markers (diameter: 0.9 mm, length: 7 mm), implanted under TRUS guidance, which were used for assessment of inter-fraction needle motion, assessed by pelvic x-ray before each fraction. In case of dislocation all applicators were repositioned before the next fraction until complete agreement was found with the reference x-ray. A video capture software (Nucletron) was used to transfer the US images to the Plato® treatment planning software. The prostate gland and the organs at risk (OAR) i.e. prostatic urethra and rectum were contoured. The dose to the rectum and urethra was evaluated using dose volume histogram (DVH). The contouring of the urethra and the rectum was based on the ultrasound image: the urethra was delineated from the apex of the prostate to the base of the bladder, the rectum was defined as the rectum sector on the ultrasound image along the planning target volume (PTV) including the muscularis propria.

The prostate without safety margins was defined as the PTV. Based on DVH analysis, the quality of plans and implants was evaluated using the following indicators: The PTV V_100_ (% of PTV receiving ≥100% of the PD) and D_90_ (dose delivered to 90% of the PTV), both of which were recorded. To assess dose exposure of OAR, the absolute values of V_70_, V_80_ of the rectum and V_100_, V_120_, V_125,_ V_150_ of the prostatic urethra were determined (volume of the OAR that received a dose ≥70%, 80% or 100%, 120%, 125%, 150% of the PD, respectively). Additionally, the maximum dose D_1_ of the rectum and urethra (defined as the dose that encompass ≥ 1% of the rectal or urethral volume) and the number of needles used was recorded. The dosimetry optimization goals were to achieve a most conformal as possible coverage of the prostate taking into consideration sometimes an under dosage in apex and base regions and at the same time optimizing the dwell times to prevent locally very high dose values. A hard constraint was: PTV V_100_ > 90%. The max dose on the rectum was required to be less then 80% (V_80_ = 0) of the prescribed dose and the maximal dose for the urethra less than 150% (V_150_ = 0) of the prescribed dose. The total dose was prescribed to the 100% isodose covering the PTV. A high-dose-rate afterloading system (Nucletron) with a ^192^Ir stepping source was used.

### Follow-up protocol

Follow-up visits were arranged 2–4 weeks after completion of HDR-BT and every 3 to 6 months for the first 2 years and annually thereafter with a digital rectal examination and a serum PSA level obtained at each visit. Patients alternated follow-up visits between their urologist and radiation oncologist. None of the patients were lost to follow-up.

### Toxicity scoring

The symptoms dysuria, incontinence, retention, frequency/urgency, hematuria, diarrhea, rectal pain and rectal bleeding were graded using the common terminology criteria for adverse events from the National Cancer Institute (CTCAE version 3.0). Erectile dysfunction (ED) was scored based on clinical symptoms as follows: grade 0 = no symptoms of ED; grade 1 = decreased erectile function but still able to perform sexual intercourse; grade 2 = decreased erectile function not able to perform intercourse; grade 3 = no erections. Late toxicity was defined as symptoms who increased over baseline and occurred within 3 months after the end of treatment. Late toxicity at last follow-up visit was noted and termed “last late toxicity”; this was done to assess whether the late toxicity was persistent or was transient.

### Biochemical failure

Biochemical recurrence-free survival (bRFS) was assessed according to the Phoenix criteria, defining a biochemical failure as a PSA rise of 2 ng/mL or more above the nadir PSA.

### Statistical analysis

Descriptives include absolute and relative frequencies for categorical variables and the median and interquartile range (IQR) for quantitative variables.

The primary objective was the occurrence of late grade 3 GU toxicity and secondary objectives included bRFS and late gastrointestinal (GI) toxicity. Late GU toxicity (grade 0–2 vs. grade 3) was grouped according to the number of grade 3 events observed and compared with clinical and dosimetric factors using univariate and multivariate Cox regression analyses. Actuarial toxicity-free survival and bRFS rates were calculated using the Kaplan Meier method. Statistical significance was considered on a two-sided significance level (α) of = 0.05. Statistical analysis was performed with SPSS® version 19.0 (SPSS Inc., Chicago, IL).

## Results and discussion

Patient characteristics are summarized in Table 1. The pre-implant prostate size was 40 cm^3^ (range, 24–62 cm^3^), however this information was only available for 17/36 patients. Dosimetric characteristics are summarized in Table 2. The median follow-up was 6.9 years (range, 1.5–8.0 years).

**Table 1:** Patient characteristics

**Total patients**	**(n = 36)**
Age, median (IQR), years	63 (10)
Tumor classification	
cT1	27 (75.0%)
cT2	9 (25.0%)
Gleason Score	
3-5	8 (22.2%)
6	23 (63.9%)
7	5 (13.9%)
Pre-treatment PSA	
≤10 ng/mL	31 (86.1%)
>10 ng/mL	5 (13.9%)
Percent positive biopsies	
≤ 50%	17 (47.2%)
> 50%	14 (38.9%)
Unknown	5 (13.9%)
Risk group	
Low	28 (77.8%)
Intermediate	8 (22.2%)

*Abbreviations*: *PSA* prostate-specific antigen, *IQR* interquartile range.

**Table 2:** Dosimetric characteristics

**Dosimetric measure**	**Mean (SD)**	**Median (IQR)**
PTV V_100_ (%)	93.5 (5.7)	95.5 (8.7)
PTV D_90_ (Gy)	38.8 (7.3)	41.2 (5.3)
Urethra V_100_ (cm^3^)	0.46 (0.18)	0.44 (0.18)
Urethra V_120_ (cm^3^)	0.16 (0.14)	0.15 (0.23)
Urethra V_125_ (cm^3^)	0.08 (0.09)	0.03 (0.14)
Urethra V_150_ (cm^3^)	0.0 (0.0)	0.0 (0.0)
Rectal V_70_ (cm^3^)	0.1 (0.1)	0.08 (0.005)
Rectal V_80_ (cm^3^)	0.006 (0.02)	0.00 (0.005)

*Abbreviations*: *IQR* interquartile range, *PTV* planning target volume, *PTV V*_*100*_ percentage of PTV receiving ≥100% of prescribed dose, *D*_*90*_ dose administered to 90% of the PTV, *urethral V*_*100*_*-V*_*150*_ absolute urethral volume that received a dose ≥100%, 120%, 125%, 150% of the prescribed dose, *rectal V*_*70*_*-V*_*80*_ absolute rectal volume that received a dose ≥70% or 80%, of the prescribed dose.

### Late genitourinary and gastrointestinal toxicity

**Figure 2:**
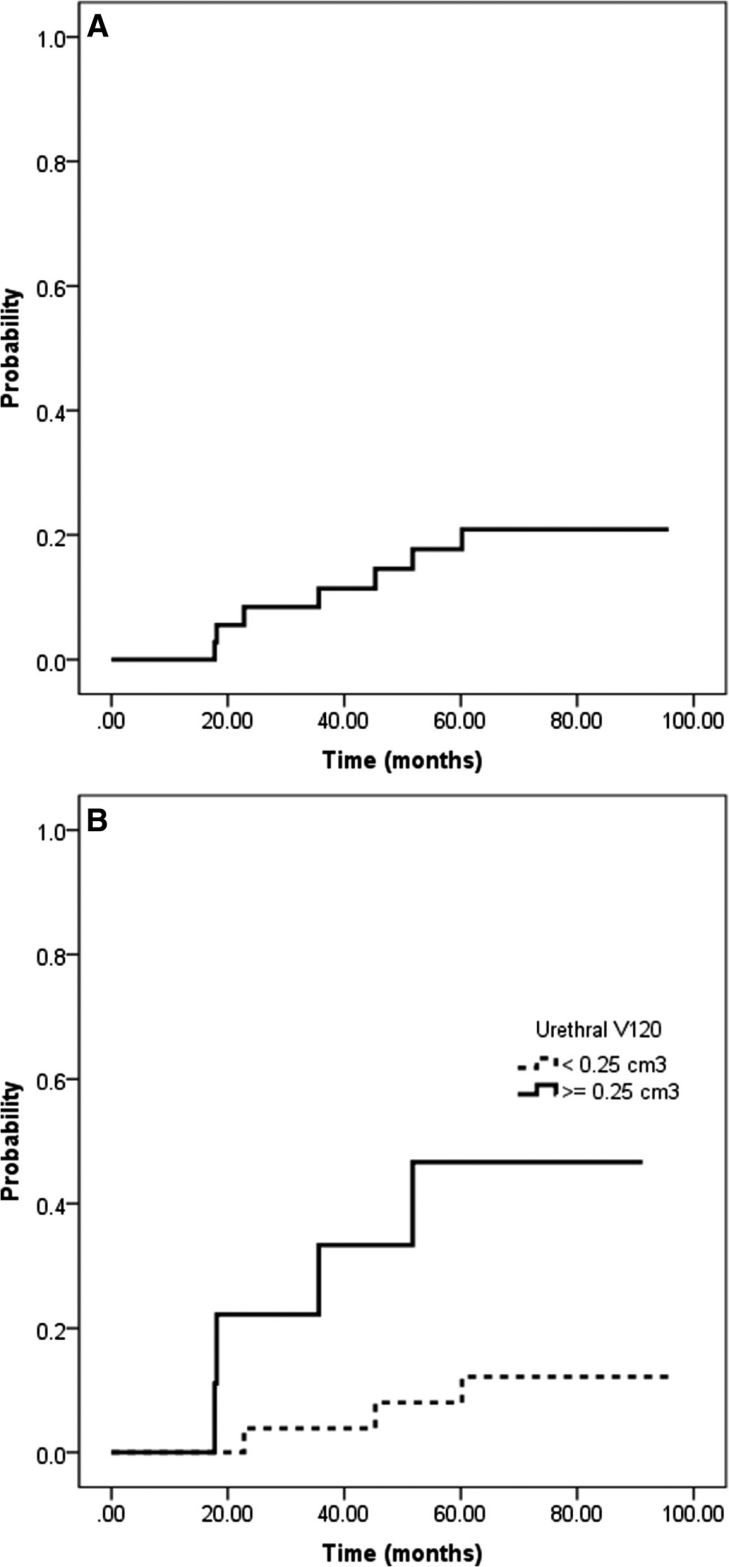
**Kaplan Meier plots showing the probability of late grade 3 genitourinary toxicity for all patients (A) and stratified according to the urethral V**_**120 **_**(B).**

Baseline symptoms, acute toxicity and early late toxicity was previously described. Regarding GU baseline symptoms, these were completely absent in around 53%, and were of grade 1 in 47% of the patients. GI baseline symptoms were absent in around 91% and of grade 1 and 2 in 3% and 6% of the patients, respectively [9]. Late GU toxicity was of grade 2 in 10 (28%) and of grade 3 in 7 (19%) (Table 3). The median time from RT completion to the occurrence of late grade 3 GU toxicity was 77 months (IQR, 41.45 months). The 5 year probability of late grade 3 GU toxicity was 17.7% (Figure 2A). All 7 patients who developed late grade 3 GU toxicity underwent an urological intervention. Six patients had a bulbar urethral stricture requiring urethral dilatation, in 4 patients an additional TUR-P was performed. The remaining patient presented with acute urinary retention and received a foley catheter and underwent a TUR-P shortly thereafter. Late GU toxicity decreased as time from treatment completion elapsed. At the last follow-up visit late grade 3 GU toxicity was not observed (Table 3).

**Table 3:** Late genitourinary toxicity

		**Late**^**†**^	**Last late**^**§**^
**Symptom**	**Grade**	**n (%)**	**n (%)**
Dysuria	0	23 (64)	36 (100)
	1	8 (22)	0 (0)
	2	5 (14)	0 (0)
Incontinence	0	29 (81 )	33 (92)
	1	7 (19)	3 (8)
Retention	0	19 (53)	27 (75)
	1	3 (9)	6 (17)
	2	7 (19)	3 (8)
	3	7 (19)	0 (0.0)
Frequency/urgency	0	16 (44)	25 (70)
	1	13 (36)	7 (19)
	2	6 (17)	4 (11)
	3	1 (3)	0 (0.0)
Hematuria	0	28 (78)	34 (94)
	1	4 (11)	1 (3)
	2	4 (11)	2 (3)
Highest GU*	0	11 (31)	21 (58)
	1	8 (22)	9 (25)
	2	10 (28)	6 (17)
	3	7 (19)	0 (0)

*Abbreviations*: *GU* genitourinary, *The highest toxicity in a patient was counted as a single event, ^†^> 3 months after completion of therapy, ^§^Incidence of late toxicity at last follow-up visit.

No patient had late grade 2 or 3 GI toxicity at the last follow-up visit all grade 1 late GI toxicities were ameliorated (Table 4).

**Table 4:** Late gastrointestinal toxicity

		**Late**^**†**^	**Last late**^**§**^
**Symptom**	**Grade**	**n (%)**	**n (%)**
Diarrhea	0	33 (92)	36 (100)
	1	3 (8)	0 (0)
	2	0 (0)	0 (0)
Rectal pain	0	36 (100)	36 (100)
	1	0 (0.0)	0 (0.0)
Rectal bleeding	0	36 (100)	36 (100)
	1	0 (0)	0
Highest GI*	0	33 (92)	36 (100)
	1	3 (8)	0 (0)
	2	0 (0)	0 (0)

*Abbreviations*: *GI* gastrointestinal, *The highest toxicity in a patient was counted as a single event, ^†^> 3 months after completion of therapy, ^§^Incidence of late toxicity at last follow-up visit.

### Factors associated with genitourinary toxicity

There was no association between late ≥ grade 2 GU toxicity and clinical or dosimetric factors. However the occurrence of late grade 3 GU toxicity was associated with clinical and dosimetric variables after univariate Cox regression analysis (Table 5). Late grade 3 GU toxicity was associated with the urethral volume (*p* = 0.001) and the urethral V_120_ (urethral volume receiving ≥120% of the prescribed dose; *p* = 0.0005) after multivariate Cox regression (Table 4). The quartiles for the urethral V_120_ were 25% = 0.02 cm^3^; 50% = 0.15 cm^3^ and 75% = 0.25 cm^3^. The probability of late grade 3 GU toxicity stratified to the 75% quartile of the urethral V_120_ is depicted in Figure 2B. Significant correlations between the urethral V100, V120 and V125 and increased PTV V100 and D90, respectively have previously been described [9].

**Table 5:** Univariate and multivariate associations with late grade 3 genitourinary toxicity

**Variables**	**Associated level**	**Late grade 3 GU toxicity**	
		**Hazard ratio (95% CI)**	**p-value**
*Univariate analysis*			
Age (years)	Continuous	1.031 (0.921-1.154)	0.596
Risk group*	Intermediate	0.659 (0.079-5.481)	0.699
Prostate volume^#^	Continuous	0.928 (0.835-1.032)	0.170
Number of Applicators	Continuous	1.247 (0.906-1.718)	0.176
PTV volume (cm^3^)	Continuous	1.014 (0.974-1.056)	0.504
PTV V_100_ (%)	Continuous	>1000 (0.000- > 1000)	0.371
PTV D_90_ (Gy)	Continuous	83.476 (0.019- > 1000)	0.301
Urethral volume (cm^3^)	Continuous	41.143 (2.754-614.621)	0.007
Urethral D_1_ (Gy)	Continuous	6.946 (0.000- > 1000)	0.692
Urethral V_100_ (cm^3^)	Continuous	183.29 (6.785- > 1000)	0.002
Urethral V_120_ (cm^3^)	Continuous	926.461 (2.653- > 1000)	0.022
Urethral V_125_ (cm^3^)	Continuous	405.747 (0.249- > 1000)	0.111
Acute grade 3 GU toxicity	Yes	7.846 (0.876-70.307)	0.066
*Multivariate analysis*			
Urethral volume (cm^3^)	Continuous	338.940 (9.502- > 1000)	0.001
Urethral V_100_ (cm^3^)	Continuous	-	
Urethral V_120_ (cm^3^)	Continuous	5778.111 (14.398- > 1000)	0.005
Acute grade 3 GU toxicity	Yes	-	

*Abbreviations*: *GU* genitourinary, *PTV* planning target volume, *PTV V*_*100*_ percentage of PTV receiving ≥100% of prescribed dose, *D*_*90*_ dose administered to 90% of the PTV, *Urethral V*_*100*_*-V*_150_ absolute urethral volume that received a dose ≥100%, 120%, 125%, 150% of the prescribed dose, *Urethral D*_*1*_ maximal dose that encompass 1% of the urethral volume, *according to the National Comprehensive Cancer Network **(**NCCN), ^#^pre-treatment, *ADT* androgen deprivation therapy.

### Erectile function

Of the 31 patients who did not receive ADT, 20 (65%) had no symptoms of ED before treatment and 8 (26%) had decreased erectile function but were still able to perform sexual intercourse. The remaining three patients had decreased erectile function and were unable to perform intercourse. At the latest follow-up visit 21 of these 28 patients (75%) were able to perform intercourse. Thus, the erectile function preservation rate was 75% in patients without ADT. Of these 21 sexually active men 7 patients received oral therapy with a phosphodiesterase type 5 inhibitor.

### Biochemical outcome

During follow-up one patient with low-risk disease who did not receive ADT experienced biochemical recurrence after 56 months. The 5 year bRFS according to the Phoenix criteria was 97%. Overall the median PSA nadir value was 0.1 ng/mL (IQR, 0.235 ng/mL) and time to nadir was 61.6 months (IQR, 42.24 months). During follow-up two patients died, both in the absence of biochemical recurrence, thus the crude overall survival rate was 94%.

This report describes the 5-year late toxicity and bRFS outcome, based on a median follow-up of almost seven years, in association with DVH parameters in patients with low and intermediate risk prostate cancer treated by HDR-BT monotherapy.

We are aware of 5 cohorts of patients treated by HDR-BT as a monotherapy using more than 3 fractions and reporting on late toxicity and biochemical control data (Table 6) [1-5].

**Table 6:** Study results on HDR-BT as a monotherapy using more than 3 fractions

**Study (reference)**	**n**	**M. follow-up (vrs)**	**Risk**	**HDR schedule**	**ADT**	**Toxicity score**	**G2 GI late toxicity**	**G3 GI late toxicity**	**G2 GU late toxicity**	**G3 GU late toxicity**	bRFS*
Demanes (1)	298	5.2	L 81%, I 18%, H 1%	6 × 7 Gy (53%)	24%	CTCAE 3.0	<1%	<1%	10%	3%	97%
				4 × 9.5 Gy (47%)							
Zamboglou (2)	718	4.4	L 55%, I 25%, H 20%	4 × 9.5 Gy (68%)	21.4%	CTCAE 3.0	~1%	1.6%	~20%	3.5%	94%
				3 × 11.5 Gy (32%)							
Yoshioka (3)	112	5.4	L 13%, I 26%, H 61%	9 × 6 Gy	84%	CTCAE 3.0	12%^#^	3%^#^	12%^#^	3%^#^	83%
Hoskin (4)	197	0.5-5	L 4%, I 52%, H 44%	4 × 8.5 Gy (15%)	80%	RTOG	1%	4-13%	20-30%	3-14%	95%
				4 × 9 Gy (13%)							
				3 × 10.5 Gy (55%)							
				2 × 13 Gy (17%)							
Rogers (5)	284	2.7	I 100%	6 × 6.5 Gy	16%	RTOG	0%	0%	1.8%	0.7%	83%
Present study	36	6.9	L 78%, I 22%	4 × 9,5 Gy	14%	CTCAE 3.0	0%	0%	28%	19%	97%

*Abbreviations*: *M* median, *G* grade, *GU* genitourinary, *GI* gastrointestinal, *L* low, *I* intermediate, *H* high, *bRFS* biochemical recurrence-free survival, *according to the Phoenix criteria, ^#^genitourinary and gastrointestinal toxicity not discriminated.

Meanwhile, further reports on extreme HDR-BT hypofractionation using only 3 [6] 2 [7] or only 1 fraction [8] became available with promising results regarding cancer control and acute and early late toxicity. However, due to their relatively short median follow-up of < 40 months, these results must be regarded as preliminary.

Compared to the mentioned literature the biochemical control rate of 97% in our low-intermediate-risk patient cohort was excellent, the erectile function preservation rate of 75% after almost 7 years of follow-up was encouraging and late GI toxicity was negligible. However the observed late GU grade 2 and 3 toxicity rates of 28 and 19% were in the upper range of published studies. Remarkably, some of the grade 2 events were transient and resolved until the end of follow-up were the incidence of late grade 2 toxicity was 17%. Moreover, all grade 3 events were successfully managed by urethral dilatation followed by a TUR-P in 4 of 6 cases. We found an association between late grade 3 toxicity and the urethral V_120_ after multivariate analysis. The association between the dose to the prostatic urethra and the occurrence of bulbar urethral strictures might be indirectly caused by radiation of vascular support of the urethra. Uncertainties of the delineation of the urethra without use of hydrogel might also have contributed to excess dose to the urethra.

We used only one implant for the 4 fractions applied over 2 days and controlled interfractional movement of the applicators using pre-implanted gold markers and orthogonal conventional X-rays. As previously described significant applicator shifts were detected and corrected and these shifts were not associated with the occurrence of GU toxicity [10]. However, we cannot exclude that some applicator shifts were not recognized contributing to impaired dosimetry and subsequently late GU toxicity. Also, during planning the rectal ultrasound probe was in place with legs up in lithotomy position while treatment was delivered with legs straight and without ultrasound probe which has been described as unfeasible by others [11]. The conduction of 2 separate implants might reduce late GU toxicity and improved image-guided procedures in brachytherapy could help to limit these effects, in future. Moreover, patients were not assessed with the International Prostate Symptom Score (IPSS) at baseline. It might be that exclusion of patients with significant baseline symptoms according to the IPSS would have reduced the need for urological interventions in our cohort. Also the use of Cox regression analysis is limited because of the small sample size and low number of events. As a note of caution, we have also to emphasize that the median follow-up time of present study is the longest reported so far. It might well be, that the grade 3 toxicities will increase in other patients cohorts with longer follow-up time. Additionally, it must be acknowledged that most of the low-risk patients in our cohort might alternatively have been treated by an active surveillance approach which might be associated with comparable tumor control rates but less GU toxicity [12] – a concept, however, which was not well established at that time.

## Conclusions

Late grade 3 GU toxicity occurred in 19% of patients. Proper patient selection, reduction of urethral dose (V120 below 0.25 cm^3^) along with a rigourous quality management including proper replanning or even online planning at each fraction might reduce this rate. Late GI toxicity after this treatment was negligible and the erectile function preservation rate and the 5 year bRFS was excellent.

## Abbreviations

HDR-BT: High-dose-rate brachytherapy; PD: Prescribed dose; ADT: Androgen deprivation therapy; GU: Genitourinary; GI: Gastrointestinal; bRFS: Biochemical recurrence-free survival; TRUS: Transrectal ultrasound; CT: Computed tomography; MRI: Magnetic resonance imaging; GnRH: Gonadotropin releasing hormone analogue; OAR: Organs at risk; DVH: Dose volume histogram; PTV: Planning target volume; ED: Erectile dysfunction; CTCAE: Common terminology criteria for adverse events; IQR: Interquartile range; IPSS: International prostate symptom score.

## Competing interests

The authors declare that there are no financial disclosures or conflict of interest that could be perceived as prejudicing the impartiality of the research reported.

## Authors’ contributions

Each author had participated sufficiently in the work to take public responsibility for appropriate portions of the content. PG and DMA designed the study. PG and SLO performed the statistical analysis. SLO, NC and PG collected the data and together with CAR, BI, GNT and DMA interpreted the data. The manuscript was written by SLO and PG, all other authors helped and finally approved the final manuscript.
